# Predicting the need for supportive services after discharged from hospital: a systematic review

**DOI:** 10.1186/s12913-020-4972-6

**Published:** 2020-03-04

**Authors:** Daniel M. Kobewka, Sunita Mulpuru, Michaël Chassé, Kednapa Thavorn, Luke T. Lavallée, Shane W. English, Benjamin Neilipovitz, Jonathan Neilipovitz, Alan J. Forster, Daniel I. McIsaac

**Affiliations:** 1 Department of Medicine, Division of General Internal Medicine, The Ottawa Hospital, Civic Campus, 1053 Carling Avenue, Ottawa, Ontario ON K1Y 4E9 Canada; 2 Department of Medicine, University of Ottawa, Ottawa, Ontario Canada; 3 Clinical Epidemiology Program, Ottawa Hospital Research Institute, Ottawa, Ontario Canada; 4 Department of Medicine (Critical Care), University of Montreal Hospital, Montreal, Quebec, Canada; 5 School of Epidemiology and Public Health, University of Ottawa, Ottawa, Ontario Canada; 6 Institute for Clinical Evaluative Sciences, Toronto, Ontario Canada; 7 Division of Urology, Department of Surgery, University of Ottawa, Ottawa, Ontario Canada; 8 Performance Measurement, The Ottawa Hospital, Ottawa, Ontario Canada; 9 Departments of Anesthesiology & Pain Medicine, The Ottawa Hospital, University of Ottawa, Ottawa, Ontario Canada

**Keywords:** Discharge, Residential facilities, Assisted living, Home care

## Abstract

**Background:**

Some patients admitted to acute care hospital require supportive services after discharge. The objective of our review was to identify models and variables that predict the need for supportive services after discharge from acute care hospital.

**Methods:**

We performed a systematic review searching the MEDLINE, CINAHL, EMBASE, and COCHRANE databases from inception to May 1st 2017.

We selected studies that derived and validated a prediction model for the need for supportive services after hospital discharge for patients admitted non-electively to a medical ward. We extracted cohort characteristics, model characteristics and variables screened and included in final predictive models. Risk of bias was assessed using the Quality in Prognostic Studies tool.

**Results:**

Our search identified 3362 unique references. Full text review identified 6 models. Models had good discrimination in derivation (c-statistics > 0.75) and validation (c-statistics > 0.70) cohorts. There was high quality evidence that age, impaired physical function, disabilities in performing activities of daily living, absence of an informal care giver and frailty predict the need for supportive services after discharge. Stroke was the only unique diagnosis with at least moderate evidence of an independent effect on the outcome. No models were externally validated, and all were at moderate or higher risk of bias.

**Conclusions:**

Deficits in physical function and activities of daily living, age, absence of an informal care giver and frailty have the strongest evidence as determinants of the need for support services after hospital discharge.

**Trial registration:**

This review was registered with PROSPERO #CRD42016037144.

## Introduction

Patients discharged from hospital often have impaired ability to perform instrumental activities of daily living (IADLs - e.g. meal preparation, managing finances or house work) and activities of daily living (ADLs – e.g. dressing, bathing and toileting) [1]. While many patients eventually recover to their pre-hospital level of function, 30–50% never do [2]. This is especially true for frail elderly patients with multiple comorbidities, who often require community based supportive services, or transition to a long-term care facility to meet their care needs [3].

Matching a patient’s need for assistance to appropriate support services is important as it can minimize the risk of unplanned readmissions and adverse events post discharge [4]. However, assessing the need for assistance requires resources. Early prediction of a patient’s need for support services after hospital discharge could improve patient care and reduce costs. This could be achieved by completing assessments of functional status early in the hospital course, in order to facilitate timely coordination of community services or alternate discharge locations.

Several models have been proposed to predict the location of discharge or the level of supportive services required after discharge. However, there is only one review of tools to predict location of discharge and it only used qualitative synthesis to describe them [5]. No review has compared validated models that predict the need for supportive services after discharge to home or an institutional setting. Given the importance of identifying individuals in need of post-discharge services, and the heterogeneity of models and predictors in the literature to date, our primary objective was to identify validated models that predict the need for any support service after discharge from a non-elective general medicine hospitalization, and evaluate the strength of evidence for predictor variables.

## Methods

### Study registration

This systematic review is reported according to the Preferred Reporting Items for Systematic reviews and Meta-analyses (PRISMA) checklist with the protocol published and registered at PROSPERO (Protocol #CRD42016037144) [6, 7].

### Eligibility criteria

We included retrospective and prospective studies that derived and validated a predictive model for the need for supportive services for adults (≥18 years of age) discharged from a non-elective general medical inpatient ward or medical sub-specialty ward. We defined supportive services as medical care or formal assistance with IADLS or ADLs at home or in an institutional environment (e.g., skilled nursing facility). We excluded studies of patients admitted to rehabilitation hospitals and studies where patients were discharged to a rehab hospital or long-term acute care hospital that was not the final discharge destination. We limited the review to validated models to avoid variables that have spurious associations with the outcome due to random chance or overfitting [8]. Internal validation will not detect all over fitting but in order to increase the number of studies in our review we considered a model validated if it had at a minimum performed internal validation.

### Literature search and information sources

Our search strategy was designed in an iterative process with the assistance of a medical information specialist. We used medical subject headings (MeSH) terms and free text terms representing the included study types, population, and outcomes. To account for geographical variations in describing supportive services we included terms such as home care, skilled nursing facility, care home, and nursing home (see Additional file 1). Our search strategy formatted for MEDLINE can be found in Additional file 1. We searched the MEDLINE, CINAHL, EMBASE, and COCHRANE databases from inception to May 1st 2017 with no limitation based on language. We hand searched the reference lists of published systematic reviews, and eligible studies. Duplicates were removed prior to stage 1 screening.

### Study selection and data collection

The title and abstract of all references were screened for eligibility independently by two reviewers (DIM, JN, or BN). Studies written in a language other than English were translated using Google© Translate prior to screening [9]. Full-text articles for all potentially eligible papers were obtained and reviewed in duplicate by two independent reviewers. Data extraction was performed independently and in duplicate with all eligibility and extraction disagreements resolved by consensus. Screening and data extraction were performed with Distiller SR® (Ottawa, Canada).

The data extraction was guided by the Checklist for critical Appraisal and data extraction for systematic Reviews of prediction Modelling Studies (CHARMS) [10]. Our data extraction form was pilot tested on for 2 studies, modified and then used for the remaining studies. All data extraction was performed in duplicate with disagreements resolved by consensus. We extracted study characteristics including setting, design, prognostic variable collection timeframe, and sample size for the derivation and validation cohorts. We collected patient characteristics including the mean age of participants, most common admitting diagnoses, and predictor variables used in model development. We collected model characteristics including variable selection method, method of screening variables for inclusion, and variables included in the final model, discrimination and calibration.

### Synthesis of results

Our primary analysis was a narrative description of models that predict the need for supportive services after discharge, and the predictor variables included in the models.

### Risk of bias and quality of evidence

Two reviewers (DK and DIM) independently used the Quality In Prognosis Studies (QUIPS) tool to assess the methodological quality of each study with disagreements resolved by discussion and consensus [11].

The quality of evidence for each predictor variable was summarized using the Grading of Recommendations Assessment, Development and Evaluation (GRADE) tool that has been adapted for use in narrative systematic reviews of prognostic studies [12]. Predictor variables that were associated with the outcome in a single study were not included in the strength of evidence analysis.

### Causal pathway creation

We constructed a causal pathway using the predictor variables identified in our review, informed by the concepts of directed acyclic graphs [13]. Directed acyclic graphs are a graphical method for representing causal relationships. Predictor variables that were associated with the outcome only in univariable analysis but not in multivariate analyses were assumed to be confounders or to be indirectly causing the outcome through a more direct effect mediator [14]. The causal pathway construction was guided by the review results and by the expert knowledge of the authors in an iterative process.

### Role of the funding source

No funding source.

## Results

**Fig. 1:**
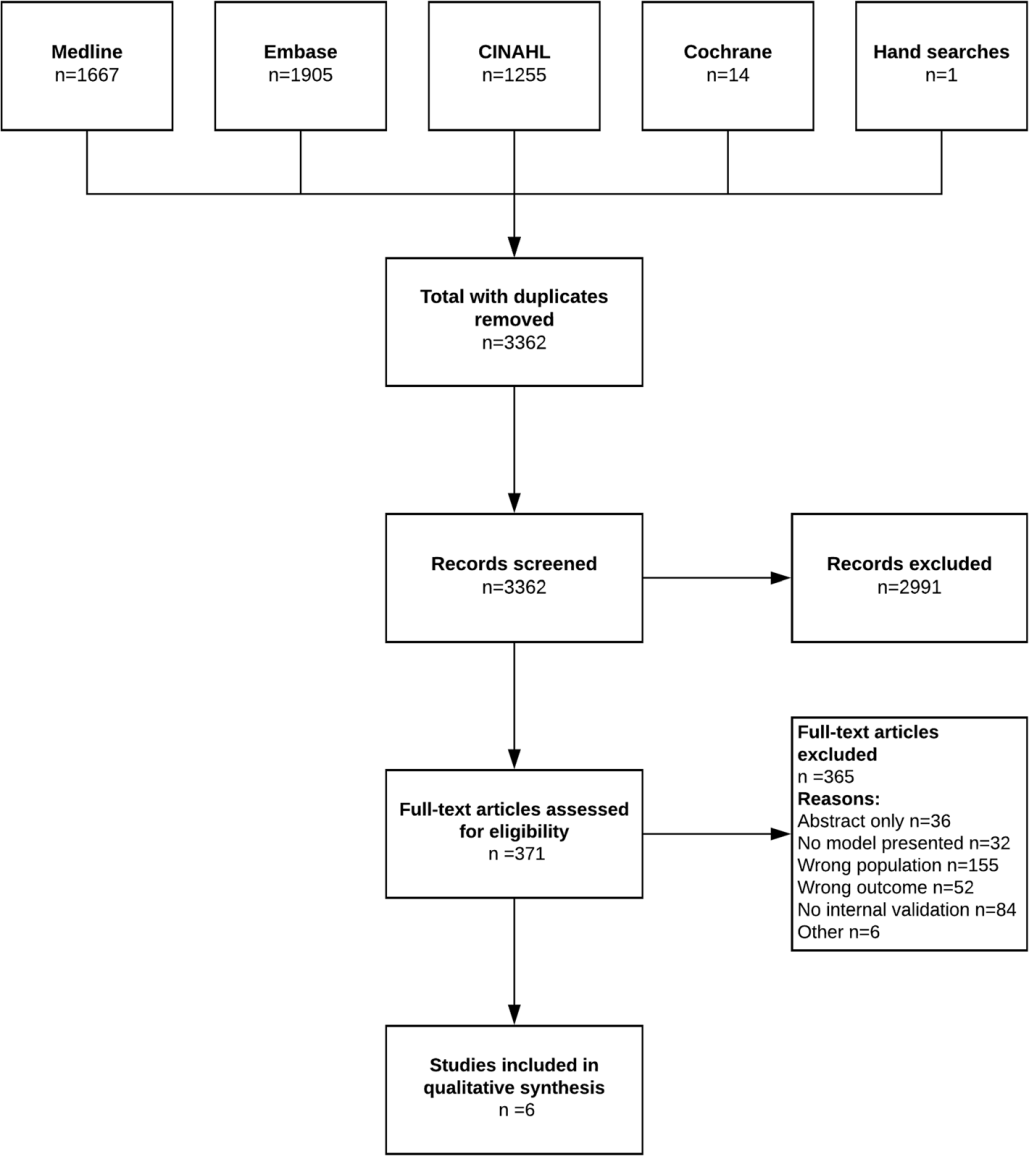
Search and Selection Process

Our predefined search strategy identified 3361 titles; none were identified through searching reference lists of published systematic reviews, and review of included study reference lists identified 1 more. The screening process and reasons for exclusion at each step are presented in Fig. 1.

We identified 6 studies describing 6 prediction models that that met our inclusion criteria, which included 7075 patients [15–20]. Four studies were from North America, one from Australia, and one from Europe. Four included general medicine patients and two included specific subpopulations that could be cared for on a general medical ward (stroke patients and patients with advanced cirrhosis) (Table 1).

**Table 1:** Studies

Study	Participants and Setting	Design	Sample size derivation cohort	Validation type and sample size	Mean age of derivation cohort	Most common admitting diagnoses	Prognostic data collection timeframe^a^	Outcome
Fairchild et al., 1998 [15]	General medicine patients in an urban teaching hospital	Single center prospective cohort	387	Internal, *n* = 327	55	Chest pain	< 24 h	Use of post-discharge medical services
						Heart failure and shock		
						Bronchitis and asthma with complications		
Simonet et al., 2008 [20]	General medicine patients in a teaching hospital	Single center prospective cohort	349	Internal, *n* = 161	65	Not provided	< 24 h	Discharge to a post-acute care facility
Mehta et al., 2011 [16]	General medicine patients in a teaching hospital	Multicenter prospective cohort	885	External, *n* = 753	78	Chronic lung disease	< 24 h	Need for ADL support
						Peripheral vascular disease		
						Congestive heart failure		
Stineman et al., 2014 [18]	Veterans hospitals stroke patients	Multicenter retrospective cohort	3909	Internal, *n* = 2606	69	Stroke	Throughout hospitalization	Home discharge
Tapper et al., 2015 [19]	Liver transplant unit in an academic hospital	Single center retrospective cohort	490	Internal, *n* = 244	57	Encephalopathy	< 24 h	Discharge to rehabilitation
						Ascites		
						Gastrointestinal bleeding		
Basic et al., 2015 [17]	Patients admitted under geriatricians with geriatric issues	Single Center Prospective Cohort	1055	Internal *n* = 1070	83	Dementia	During the Hospitalization	New Admission to a Nursing Home
						Delirium		
						Deconditioning		

^a^Relative to time of admission

The mean age of patients in the derivation cohorts ranged from 55 to 83 years.

### Risk of Bias

The risk of bias for included studies was moderate to high for most studies (Table 2).

**Table 2:** Risk of Bias Assessment

Study	Study participation	Study attrition	Prognostic factor measurement	Outcome measurement	Study confounding	Statistical analysis and reporting	Overall
Tapper et al.	Moderate	Low	Moderate	Moderate	Moderate	Low	Moderate
Stineman et al.	High	Low	Moderate	Moderate	Moderate	Low	High
Fairchild et al.	High	High	Moderate	Moderate	Moderate	Moderate	High
Simonet et al.	Moderate	Moderate	Low	Low	Low	Low	Moderate
Basic et al.	Low	Low	Moderate	Low	Moderate	Moderate	Moderate
Mehtah et al.	High	Low	Low	Low	Moderate	Low	Moderate

For each category low risk of bias is defined as: **Study participation**: The study sample represents the population of interest on key characteristics, **Study Attrition**: Loss to follow-up is not associated with key characteristics, **Prognostics Factor Measurement**: Factors are adequately measured, **Outcomes Measurement**: Outcome of interest is adequately measured, **Study Confounding:** Important potential confounders are appropriately accounted for, **Statistical Analysis and Confounding**: The statistical analysis is appropriate for the design of the study, **Overall:** Majority of criteria met. Little or no risk of bias.

Inadequate description of the source population’s inclusion and exclusion criteria was found in 5 of 6 included studies.

### Prediction models

Four studies used univariable analysis to screen for predictors to be used for modeling while the other two studies used clinical reasoning to select predictor variables. To build the final model, five prediction models used various automated selection algorithms while one included all the variables that were hypothesized to be predictive (Table 3).

**Table 3:** Models

Study	Outcome	Number (%) with outcome	Variable screening method	Variable selection method	Variables included in final model	Discrimination (derivation)	Discrimination (validation)	Calibration	Other measures of model performance
Fairchild et al., 1998 [15]	Use of post-discharge medical services	134 (35)	Univariable analysis	Selection algorithm	Age > 65, SF-36 Physical administered on admission < 50, SF-36 Social < 15	AUC = 0.75	AUC = 0.70	N/A	N/A
Simonet et al., 2008 [20]	Discharge to a post-acute care facility	104 (30)	Univariable analysis	Selection algorithm	Number of medically active conditions on admission, Inability of patient’s partner to provide home help, Number of IADL and ADL disabilities, Age, Admitted via inter-hospital transfer	AUC = 0.82	AUC = 0.77	N/A	8-point cutoff: Sensitivity 0.87/Specificity 0.63, 16-point cutoff: Sensitivity 0.42/Specificity 0.91
Mehta et al., 2011 [16]	Need for IADL support	242 (27)	Univariable analysis	Best subset algorithm	Age < 80, 80–89, > 90, Dependent in > 3 IADLS prior to admission, Number of ADL dependencies at the time of admission 1, 2–3, 4–5, Metastatic cancer or stroke, Albumin < 3 g/dL, Mobility before admission	0.78	0.78	H-L *P* = 0.40	N/A
Stineman et al., 2014 [18]	Home discharge	3348 (85)	Univariable analysis	Selection algorithm	Married, Location before admission extended care, hospital, home, Functional recovery grade at discharge, discharge cognitive grade, History of liver disease, no feeding tube required, No intensive care unit admission, Mechanical ventilation during admission	0.82	0.8	H-L *P* = 0.23	
Tapper et al., 2015 [19]	Discharge to rehabilitation	199 (15)	Clinical reasoning	None (all variables included)	Gender, Age, Ethnicity, Charlson Co-morbidity Index, Admission ADL score, Admission Morse fall risk score and Braden score, Admission MELD, Admission serum sodium, Infection, Cirrhotic decompensation, Hepato-cellular carcinoma, Admitting hepatologist	AUC = 0.85	AUC = 0.77	N/A	N/A
Basic et al., 2015 [17]	New Admission to a Nursing Home	62 (5.9%)	Clinical reasoning, literature review	Backwards selection Using LR test	Clinical frailty scale 7-point, Dementia, delirium, Age, Urinary retention, Deconditioning, Seizure disorder	N/A	N/A	N/A	N/A

Four models used predictors that were available with in the first 24 h of hospital admission while two models used variables collected throughout the hospital stay. All prediction models had a binary outcome as the dependent variable; however, each study defined the outcome differently (Table 3). Four models defined the outcome as a place (home, care facility, or rehabilitation hospital) while two models defined the outcome as the need for support services after discharge. Model discrimination was generally good (range derivation C-statistics 0.75–0.85) and similar but slightly lower in the validation cohorts (range validation C-statistics 0.70–0.80) with one study not reporting discrimination statistics. Two models tested calibration with the Hosmer-Lemeshow goodness of fit test and found no evidence of poor fit. Other calibration metrics, such as calibration plots, were not reported.

### Predictors

All variables associated with the need for post discharge supportive services in predictive models are presented in Table 3. Many variables were associated with the outcome in univariable analysis in a single study but not in multivariate analyses. Variables present in 2 or more studies were assessed for the strength of evidence using the GRADE tool (Table 4).

**Table 4:** GRADE

				Univariable			Multivariate			GRADE Factors						
Prognostic Factor	# of studies	No. of participants	Phase of Investigation	+	0	–	+	0	–	Risk of Bias	Inconsistency	Indirectness	Imprecision	Moderate/Large effect size	Dose Effect	Overall Quality
Age	5	6585	3	**4**	0	0	**4**	0	0	Moderate	✘	✘	✘	✔	✔	**++++**
Impaired Physical Function^a^	4	5530	3	**3**	0	0	**4**	0	0	Moderate	✘	✘	✘	✔	✔	**++++**
ADL disabilities	3	1724	3	**3**	0	0	**3**	0	0	Moderate	✘	✘	✘	✔	✔	**++++**
Frailty ^b^	2	1545	3	**1**	0	0	**2**	0	0	Moderate	✘	✘	✘	✔	✔	**++++**
Stroke	2	4794	3	**3**	0	0	**1**	0	0	Moderate	✘	✔	✘	✔	✔	**+++**
Supportive environment prior to admission	2	4258	3	**2**	0	0	**2**	0	0	Moderate	✘	✔	✘	✔	✘	**+++**
IADL disabilities	2	1234	3	**2**	0	0	**2**	0	0	Moderate	✘	✘	✘	✔	✘	**+++**
Cognitive impairment^c^	4	6198	3	**3**	0	0	**1**	0	0	Moderate	✘	✔	✘	✘	✔	**++**
Marital status (Married) vs Other	3	5181	3	0	0	**3**	0	0	**2**	Moderate	✘	✘	✘	✘	✔	**++**
Heart Failure	2	4794	2	**2**	0	0	0	0	0	Moderate	✘	✘	✘	✘	✘	**++**
Metastatic Cancer	2	4794	2	**2**	0	0	**1**	0	0	Moderate	✘	✘	✘	✔	✘	**++**
Lives with informal care giver	2	736	3	0	0	**2**	0	0	**1**	Moderate	✘	✘	✘	✘	✘	**++**
Sex (Female)	4	5633	3	**1**	**3**	0	**1**	**1**	0	Moderate	✔	✘	✘	✘	✘	**+**
Heart Valve Disease	2	4296	3	**2**	0	0	0	0	0	**High**	✘	✘	✘	✘	✘	**+**
Comorbidities Increasing number	4	2111	3	**3**	**1**	0	0	**1**	0	Moderate	✔	✘	✔	✘	✘	**+**

^a^Physical function as measured by mobility, gait, ability to transfer or physical function scores

^b^Measured using Rockwood frailty scale, Braden risk and Morse fall risk

^c^Cognitive impairment defined as low mini-mental state score, severe cognitive impairment, dementia or cognitive stage

Strength of Evidence + very low; ++ low; +++ moderate; ++++ high. The overall quality of evidence for a factor is rated as high if it comes from explanatory research aimed at understanding causal pathways (phase of investigation 3) or moderate if it comes from prediction research aimed at identifying associations (phase of investigation 2). The quality of the evidence is then downgraded if there are study limitations, inconsistency, indirectness, imprecision or publication bias. The quality is upgraded if there is moderate or large effect size ore and exposure-response gradient identified [12].

There is high quality evidence that age, impaired physical function, ADL disabilities, and frailty increase the probability of needing supportive services after discharge. There is moderate evidence that a diagnosis of stroke, IADL disabilities and receiving supportive services prior to hospital admission increase the probability of needing supportive services after discharge. Interestingly a greater number of comorbidities was a significant predictor in univariable analyses in 3 studies but not in any multivariate analyses. Indicators of absent support at home (marital status and absence of an informal care giver) had moderate-weak evidence of predicting discharge with supportive services. Lastly several specific diagnoses (heart failure, metastatic cancer and heart valve disease) had weak to very week evidence for predicting the need for supportive services.

### Causal pathway

**Fig. 2:**
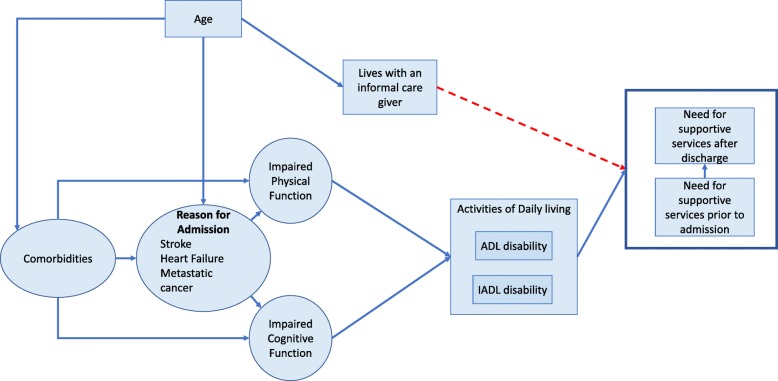
Causal pathway showing relationships between predictor variables and outcome

We constructed a causal pathway to explain the relationships between the predictor variables and the outcome (Fig. 2). Activities of daily living and the presence of a care giver at home directly affected the need for supportive services after discharge whereas age and comorbidities act by their effects on physical function and cognitive function to affect ADL disabilities. Stroke directly causes ADL and IADL disabilities while the causative impact of other specific diagnoses is less certain.

## Discussion

Our systematic review identified 6 validated models that predict the need for supportive services after discharge from a non-elective medical admission. All models had good discrimination, although there was at least a moderate risk of bias for all studies and no models were externally validated. Furthermore, other important model characteristics, such as calibration that indicates if the predicted risk is similar to the actual risk, were not reported for most models. Our GRADE analysis suggests that age, impaired physical function, ADL disabilities and frailty all have high quality evidence as factors that predict the need for supportive services after discharge.

Accurate prediction of the need for supportive services after discharge may be useful to target patients who would benefit most from discharge planning interventions. Several studies have shown that discharge planning interventions are associated with small reductions in length of stay and fewer readmissions but do not impact resource utilization or patient mortality [21]. Discharge planning interventions themselves are resource intensive and should be allocated to patients who will benefit the most [22]. Future models to predict patients who will benefit from personalized discharge planning should explore using the 15 variables identified in our review. A model building process that starts with all 15 variables we identified may result in a model with more predictive power that those we identified.

### Models

Our review included far fewer studies than other recent reviews because we excluded studies that performed no model validation. Notably none of the studies we included were externally validated calling into question the generalizability to other populations. Validation is a crucial step in model development as it assesses whether the model accurately represents the real world [8, 23]. Despite limiting our inclusion criteria to studies with validated models, several methodologic concerns remained. Only 1 model used the recommended best practice of using clinical reasoning to guide variable selection and final model building [24]. The rest of the models used univariable analysis to select variables and then an automated selection algorithm for model building. This process can result in inaccurate or false relationships between predictors and the outcome by accidentally conditioning on a collider [25]. Any model that seeks to explain the causes of an outcome needs to start with a clinical reasoning or a formal causal hypothesis so that appropriate potential causal factors and confounders can be included, thereby avoiding false or inaccurate effect estimates. Making causal hypotheses explicit is critical to furthering the field and allowing critical appraisal by peers.

### Predictors

Consistent with previous studies, we found that impairments in physical function and ADL disabilities are strong predictors of the need for supportive services after discharge [26, 27]. This is intuitive because support for ADLs is a primary service offered across the spectrum of supportive living options. Numerous medical diagnoses, illness severity scores, and comorbidity scores were associated with the need for supportive services after discharge in univariable analysis but most of them were excluded in variable selection processes. This likely occurred because comorbidities increase the need for services after discharge primarily by their impact on physical and cognitive function as illustrated in our causal pathway (Fig. 2). Once physical and cognitive function are directly measured and added to the model the other down variables that impact function are no longer significant predictors.

Marital status and living with an informal care giver were both negatively associated with the need for supportive services after discharge. Clearly, an informal caregiver may reduce the need for formal caregiving by assisting with ADLs and IADLs [28]. Informal caregivers may also impact the need for supportive services by providing emotional support and reducing loneliness [29]. Several studies in our review found a univariable association between measures of mental health and need for supportive services but the associations were not significant in final multivariate models.

Interestingly none of the included studies tested income or financial resources as predictors. The omission is likely pragmatic but considering the importance of these variables for other health outcomes we suggest that the predictive contribution of financial resources or other proxies of socioeconomic status should be considered in future derivation of models predicting need for supportive services [30]. Financial resources may not be as strong of a predictor in countries with publicly funded healthcare.

### Significance and limitations

Our findings add to the literature by distilling the key variables that predict the need for supportive services after discharge and synthesizing a cohesive theory of how each variable contributes to the need for supportive services. Based on our causal pathway, accurate assessment of ADL and IADLs of hospitalized patients may be the best method to determine if support will be needed after discharge.

Our review has several limitations. Our outcome was broadly defined as supportive services after discharge and each study defined the outcome differently. Therefore, we do not know if the predictor variables we identified apply to all types of supportive services. Another limitation is that we did not specify when each predictor variable was measured. For example, assessing ADL disability on the day of admission or 1 week into the admission will likely have different value as a predictor but we did make this distinction. Lastly, we did not search gray literature so we may have missed studies that were never published and indexed in a database.

There were also several limitations due to the available studies. None of the included studies measured function in the weeks and months prior to hospital admission or looked at how different causes of acute illness affect functional recovery. We were also unable to perform meta-analysis as each study specified a slightly different outcome.

## Conclusions

The need for supportive services post discharge can be predicted with reasonable accuracy by existing models. However, none have excellent discrimination and no model includes all variables that we identified to be associated to with needing supportive services after discharge. Our causal pathway suggests that a thorough assessment of functional status and the absence of an informal care giver directly cause a person to need supportive services. A model that includes all directly causative variables accurately measured will likely have more predictive power than existing models. Flagging patients who may not be able to return to their previous residence can facilitate communication about planning for care and may allow health care organizations use personnel resources more effectively. The primary barrier to creating such a model is the practicality of obtaining the required data in the context of a study or in real world use.

## Supplementary information

**Additional file 1.** Predicting need for supportive services. Database: Ovid MEDLINE(R) Epub Ahead of Print, In-Process & Other Non-Indexed Citations, Ovid MEDLINE(R) Daily and Ovid MEDLINE(R) < 1946 to Present> Search Strategy: Search strategy formatted for MEDLINE.

## Data Availability

All data generated or analysed during this study are included in this published article.
