# Supplementary material for: Predicting the need for supportive services after discharged from hospital: a systematic review
Source: BMC Health Serv Res. 2020 Mar 4;20:161. doi: 10.1186/s12913-020-4972-6 (PMC7057581; doi:10.1186/s12913-020-4972-6)
Supplement: Supplementary file 1 — Additional file 1. Predicting need for supportive services. Database: Ovid MEDLINE(R) Epub Ahead of Print, In-Process & Other Non-Indexed Citations, Ovid MEDLINE(R) Daily and Ovid MEDLINE(R) < 1946 to Present> Search Strategy: Search strategy formatted for MEDLINE. [file 12913_2020_4972_MOESM1_ESM.pdf]

Database: Ovid MEDLINE(R) Epub Ahead of Print, In-Process & Other Non-Indexed Citations, Ovid MEDLINE(R) Daily and Ovid MEDLINE(R) <1946 to Present> Search Strategy:

----

1 Hospitalization/ (88962)
2 (hospitali?ed or hospitali?ation).tw,kw. (183066)
3 hospital admission$.tw. (30840)
4 patient* admitted.tw,kw. (43003)
5 or/1-4 (285983)
6 Patient Discharge/ (24190)
7 (patient* adj4 discharged).tw,kw. (24795)
8 discharge.tw,kw. (151497)
9 or/6-8 (177446)
10 (outcome* adj4 hospitali?*).tw,kw. (5054)
11 9 or 10 (181360)
12 aftercare/ or (aftercare or posthospital care).tw,kw. (9264)
13 support* service*.tw. (5521)
14 Subacute Care/ (780)
15 ((subacute or postacute or post acute) adj2 (care or service$)).tw,kw. (1457)
16 Institutionalization/ (5204)
17 exp Nursing Homes/ or (nursing home* or nursing facilit$).tw,kw. (46880)
18 retirement home$.tw,kw. (226)
19 ((intermediate or residential or institutional) adj1 care).tw,kw. (5863)
20 Progressive Patient Care/ or progressive patient care.tw,kw. (1224)
21 residential facilities/ or assisted living facilities/ or homes for the aged/ (18607)
22 ((residential or assisted living) adj2 facilit$).tw,kw. (2233)
23 "old age home".tw,kw. (124)
24 care home*.tw,kw. (3041)
25 Nurses, Community Health/ or Home Nursing/ (9217)
26 (nurs$ adj2 visit$).tw,kw. (2709)
27 Hospices/ or hospice$.tw,kw. (12758)
28 Rehabilitation Centers/ or (rehabilitation adj1 (facilit$ or centre$ or center$)).tw,kw. (13335)
29 Patient Transfer/ or patient transfer$.tw,kw. (7796)
30 or/12-29 (123413)
31 (discharge destination* or discharge location*).tw,kw. (1025)
32 Risk factors/ or risk$.tw,kw. (2025120)
33 Risk Assessment/ (218491)
34 "predictive value of tests"/ or roc curve/ or (predict$ or ROC).tw,kw. (1367050)
35 Prognosis/ (440387)
36 case mix adjustment.tw,kw. (398)
37 models, statistical/ or multivariate analysis/ or "analysis of variance"/ (393475)
38 (logistic adj2 (regression or model$)).tw,kw. (208705)
39 (multivariate adj2 (model$ or analysis or regression)).tw,kw. (200334)
40 (prognostic adj5 (identif$ or indicator$ or criteria or scor$ or factor$ or model$)).tw,kw. (109645)
41 (score or scoring system or index$ or rule).ti. (99757)
42 or/32-41 (3803922)
43 5 and 11 and 30 and 42 (1560)
44 5 and 31 and 42 (269)
45 43 or 44 (1704)
46 (child/ or infant/) not adult/ (1245261)
47 45 not 46 (1667)

Database: Embase Classic+Embase <1947 to 2017 May 16> Search Strategy:

----

1 *hospital admission/ or *hospital patient/ or *hospitalization/ (62309)
2 (hospitali?ed or hospitali?ation).tw. (280284)

3 hospital admission$.tw. (46979)
4 or/1-3 (350020)
5 *hospital discharge/ (10132)
6 discharge.tw. (224005)
7 (patient* adj4 discharged).tw. (44522)
8 or/5-7 (258430)
9 (outcome* adj4 hospitali?*).tw. (7717)
10 8 or 9 (264376)
11 aftercare/ (7032)
12 (aftercare or posthospital care).tw. (4000)
13 support* service*.tw. (7037)
14 ((subacute or postacute or post acute) adj2 (care or service$)).tw,kw. (1922)
15 *Institutionalization/ or institutionali?ation.tw. (7917)
16 nursing home/ or (nursing home* or nursing facilit$).tw. (57722)
17 retirement home$.tw. (322)
18 ((intermediate or residential or institutional) adj1 care).tw. (7375)
19 progressive patient care/ or Progressive Patient Care.tw. (1045)
20 residential home/ or assisted living facility/ (8272)
21 home for the aged/ (11932)
22 ((residential or assisted living) adj2 facilit$).tw. (2731)
23 "old age home".tw. (171)
24 care home*.tw. (3497)
25 *community health nursing/ (18683)
26 (nurs$ adj2 visit$).tw. (3352)
27 home/ or *home care/ (36416)
28 hospice/ or hospice$.tw. (17459)
29 hospice$.tw. (14676)
30 (rehabilitation adj1 (facilit$ or centre$ or center$)).tw. (11638)
31 patient transfer$.tw. (1625)
32 or/11-31 (179376)
33 (discharge destination* or discharge location*).tw. (1687)
34 *risk factor/ (49697)
35 risk$.tw. (2416886)
36 *risk assessment/ (35236)
37 prediction/ or predict$.tw. (1629961)
38 predict$.tw. (1597907)
39 case mix adjustment.tw. (468)
40 multivariate analysis/ (138886)
41 (logistic adj2 (model$ or analysis or regression)).tw. (286748)
42 (prognostic adj5 (identif$ or indicator$ or criteria or scor$ or factor$ or model$)).tw. (154576)
43 scoring system/ (212836)
44 (score or scoring system or index$ or rule).ti. (128051)
45 or/34-44 (4066329)
46 4 and 10 and 32 and 45 (1806)
47 4 and 33 and 45 (268)
48 46 or 47 (1951)
49 (child/ or infant/) not adult/ (1405109)
50 48 not 49 (1905)

CINAHL

**S49**	**S47 OR S48**	**1,255**

S48	S4 AND S45 AND S46	149
S47	S4 AND S7 AND S31 AND S45	1,177
S46	TI ((discharge destination* or discharge location*)) OR AB ((discharge destination* or discharge location*))	561
S45	S32 OR S33 OR S34 OR S35 OR S36 OR S37 OR S38 OR S39 OR S40 OR S41 OR S42 OR S43 OR S44	1,008,718
S44	TI score OR TI scoring system OR TI index OR TI rule	31,639
S43	TI prognostic N5 identif* OR AB prognostic N5 identif* OR TI prognostic N5 indicator* OR AB prognostic N5 indicator* OR TI prognostic N5 criteria OR AB prognostic N5 criteria OR TI prognostic N5 scor* OR AB prognostic N5 scor* OR TI prognostic N5 factor* OR AB prognostic N5 factor* OR TI prognostic N5 model* OR AB prognostic N5 model*	14,443
S42	TI multivariate N2 model* OR AB multivariate N2 model* OR TI multivariate N2 analysis OR AB multivariate N2 analysis OR TI multivariate N2 regression OR AB multivariate N2 regression	45,663
S41	TI logistic N2 regression OR AB logistic N2 regression OR TI logistic N2 model* OR AB logistic N2 model*	61,791
S40	(MH "Multivariate Analysis")	46,722
S39	(MH "Models, Statistical")	22,394
S38	TI case mix adjustment OR AB case mix adjustment	164
S37	(MH "Prognosis+")	303,800
S36	TI ROC OR AB ROC	6,023
S35	TI predict* OR AB predict*	224,448
S34	(MH "Predictive Value of Tests")	37,295

S33	TI risk* OR AB risk*	452,520
S32	(MH "Risk Factors+") OR (MH "Risk Assessment")	196,271
S31	S8 OR S9 OR S10 OR S11 OR S12 OR S13 OR S14 OR S15 OR S16 OR S17 OR S18 OR S19 OR S20 OR S21 OR S22 OR S23 OR S24 OR S25 OR S26 OR S27 OR S28 OR S29 OR S30	111,248
S30	TI patient transfer* OR AB patient transfer*	878
S29	(MH "Transfer, Discharge")	4,426
S28	TI rehabilitation facilit* OR AB rehabilitation facilit* OR TI rehabilitation centre* OR AB rehabilitation centre* OR TI rehabilitation center* OR AB rehabilitation center*	3,672
S27	(MH "Rehabilitation Centers+")	6,825
S26	TI hospice* OR AB hospice*	9,979
S25	(MH "Hospices")	2,821
S24	TI (nurs* N2 visit*) OR AB (nurs* N2 visit*)	2,705
S23	(MH "Community Health Nursing+")	28,144
S22	TI "old age home" OR AB "old age home"	31
S21	TI residential N2 facilit* OR AB residential N2 facilit* OR TI assisted living N2 facilit* OR AB assisted living N2 facilit*	1,983
S20	TI progressive patient care OR AB progressive patient care	36
S19	(MH "Progressive Patient Care")	431
S18	TI residential care OR AB residential care OR TI intermediate care OR AB intermediate care OR TI institutional care OR AB institutional care	3,682

S17	(MH "Residential Care+")	6,397
S16	(MH "Residential Facilities")	3,705
S15	TI retirement home* OR AB retirement home*	104
S14	TI (nursing home* or care home*) OR AB (nursing home* or care home*) OR TI nursing facilit* OR AB nursing facilit*	22,536
S13	(MH "Nursing Homes+")	22,604
S12	TI subacute N2 service* OR AB subacute N2 service* OR TI postacute N2 service* OR AB postacute N2 service* OR TI post acute N2 service* OR AB post acute N2 service*	178
S11	TI subacute N2 care OR AB subacute N2 care OR TI postacute N2 care OR AB postacuteN2 care OR TI post acute N2 care OR AB post acute N2 care	1,014
S10	(MH "Subacute Care")	1,214
S9	TI (aftercare or support* service*) OR AB (aftercare or support* service*) OR TI posthospital care OR AB posthospital care	4,428
S8	(MH "After Care")	9,114
S7	S5 OR S6	57,093
S6	TI (discharge or (patient* N4 discharged) or (outcome* N4 hospitali?*))OR AB (discharge or (patient* N4 discharged) or (outcome* N4 hospitali?*))	44,697
S5	(MH "Patient Discharge+")	22,170
S4	S1 OR S2 OR S3	123,674
S3	TI (hospital admission* or patient* admitted) OR AB (hospital admission* or patient* admitted)	19,648
S2	TI hospitali?ation OR AB hospitali?ation OR TI hospitali?ed OR AB hospitali?ed	47,081

S1	(MH "Hospitalization+")	73,433

Cochrane –NHS Economic Evaluation Database : Issue 2 of 4, April 2015 = 14 results

#1 MeSH descriptor: [Hospitalization] explode all trees 14531

#2 hospitali?ed:ti,ab,kw (Word variations have been searched) 8514

#3 hospitali?ation:ti,ab,kw 21972

#4 (hospital admission or "patient* admitted"):ti,ab 9259

#5 #1 or #2 or #3 or #4 41182

#6 MeSH descriptor: [Patient Discharge] explode all trees 1366

#7 discharge:ti,ab,kw 16210

#8 (patient* near/4 discharged):ti,ab 1577

#9 #6 or #7 or #8 16942

#10 (outcome* near/4 hospitali*):ti,ab 1066

#11 #9 or #10 17795

#12 #5 and #9 6415

#13 aftercare:ti,ab,kw (Word variations have been searched) 805

#14 posthospital care:ti,ab,kw (Word variations have been searched) 45

#15 MeSH descriptor: [Subacute Care] explode all trees 18

#16 (support* near/1 service*):ti,ab,kw 412

#17 (subacute near/2 (care or service*)):ti,ab,kw (Word variations have been searched) 57

#18 (postacute near/2 (care or service*)):ti,ab,kw 19

#19 (post acute near/2 (care or service*)):ti,ab,kw 250

#20 MeSH descriptor: [Nursing Homes] explode all trees 1271

#21 (nursing home or nursing facilit*):ti,ab,kw (Word variations have been searched) 6309

#22 retirement home*:ti,ab,kw (Word variations have been searched) 43

#23 ((intermediate or residential or institutional) near/1 care):ti,ab,kw (Word variations have been searched) 739

#24 MeSH descriptor: [Progressive Patient Care] explode all trees 18

#25 ("care home" or "care homes"):ti,ab 320

#26 "progressive patient care":ti,ab,kw (Word variations have been searched) 20

#27 MeSH descriptor: [Residential Facilities] explode all trees 1658

#28 MeSH descriptor: [Assisted Living Facilities] explode all trees 45

#29 MeSH descriptor: [Homes for the Aged] explode all trees 592

#30 ((residential or assisted living) near/2 facilit*):ti,ab,kw (Word variations have been searched) 395

#31 "old age home":ti,ab,kw (Word variations have been searched) 12

#32 MeSH descriptor: [Nurses, Community Health] explode all trees 8

#33 (nurs* near/2 visit*):ti,ab,kw (Word variations have been searched) 436

#34 MeSH descriptor: [Hospices] explode all trees 41

#35 hospice*:ti,ab,kw (Word variations have been searched) 469

#36 MeSH descriptor: [Rehabilitation Centers] explode all trees 690

#37 (rehabilitation near/1 (facilit* or centre* or center*)):ti,ab,kw (Word variations have been searched) 1272

#38 MeSH descriptor: [Patient Transfer] explode all trees 150

#39 patient transfer*:ti,ab,kw (Word variations have been searched) 7578

#40 #13 or #14 or #15 or #17 or #18 or #19 or #20 or #21 or #22 or #23 or #24 or #26 or #27 or #28 or #29 or #30 or #31 or #32 or #33 or #34 or #35 or #36 or #37 or #38 or #39 17564

#41 #5 and #11 and #40 1020

#42 risk*:ti,ab,kw (Word variations have been searched) 146380

#43 MeSH descriptor: [Risk Factors] explode all trees 24411

#44 MeSH descriptor: [Risk Assessment] explode all trees 9285

#45 MeSH descriptor: [Predictive Value of Tests] explode all trees 7439

#46 (predict* or ROC):ti,ab,kw (Word variations have been searched) 66914

#47 MeSH descriptor: [Prognosis] explode all trees 137738

#48 "case mix adjustment":ti,ab,kw (Word variations have been searched) 17

#49 MeSH descriptor: [Models, Statistical] explode all trees 16178

#50 MeSH descriptor: [Multivariate Analysis] explode all trees 5295

#51 MeSH descriptor: [Analysis of Variance] explode all trees 26852

#52 (score or scoring system or index* or rule):ti 6296

#53 (prognostic near/5 identif*):ti,ab,kw (Word variations have been searched) 618

#54 (prognostic near/5 indicator*):ti,ab,kw 418

#55 (prognostic near/5 criteria*):ti,ab,kw 105

#56 (prognostic near/5 scor*):ti,ab,kw 572

#57 (prognostic near/5 factor*):ti,ab,kw 4843

#58 (prognostic near/5 model*):ti,ab,kw 468

#59 (logistic near/2 regression):ti,ab,kw 11983

#60 (logistic near/2 model*):ti,ab,kw 7664

#61 (multivariate near/2 model*):ti,ab,kw 2472

#62 (multivariate near/2 analysis*):ti,ab,kw 13356

#63 (multivariate near/2 regression):ti,ab,kw 3726

#64 #42 or #43 or #44 or #45 or #46 or #47 or #48 or #49 or #50 or #51 or #52 or #53 or #54 or #55 or #56 or #57 or #58 or #59 or #60 or #61 or #62 or #63 326495

#65 #41 and #64 506
